# Overrepresentation of South Asian ethnic groups among cases of influenza A(H1N1)pdm09 during the first phase of the 2009 pandemic in England

**DOI:** 10.1111/irv.12801

**Published:** 2020-09-01

**Authors:** Suzan C. M. Trienekens, Wendi Shepherd, Richard G. Pebody, Punam Mangtani, Paul Cleary

**Affiliations:** ^1^ Field Epidemiology Training Programme Public Health England UK; ^2^ Field Service North West, National Infection Service Public Health England UK; ^3^ North West Health Protection Team Public Health England UK; ^4^ Department of Clinical Sciences Liverpool School of Tropical Medicine Liverpool UK; ^5^ National Infection Service Public Health England UK; ^6^ Department of Infectious Disease Epidemiology London School of Tropical Medicine and Hygiene London UK

**Keywords:** epidemiology, ethnicity, influenza, pandemic, South Asian, surveillance, transmission

## Abstract

**Background:**

During the first wave of the influenza A(H1N1)pdm09 pandemic in England in 2009, morbidity and mortality were higher in patients of South Asian (Indian, Pakistani or Bangladeshi) ethnic minority groups.

**Objectives:**

This study aims to provide insights in the representation of this group among reported cases, indicating susceptibility and exposure.

**Methods:**

All laboratory‐confirmed cases including basic demographic and limited clinical information that were reported to the FluZone surveillance system between April and October 2009 were retrieved. Missing ethnicity data were imputed using the previously developed and validated South Asian Names and Group Recognition Algorithm (SANGRA). Differences between ethnic groups were calculated using chi‐square, log‐rank and t tests and rate ratios. Geographic clustering was compared using Ripley's K functions.

**Results:**

SANGRA identified 2447 (28%) of the total of 8748 reported cases as South Asian. South Asian cases were younger (*P* < .001), more often male (*P* = .002) and more often from deprived areas (*P < .001*) than cases of other ethnic groups. Time between onset of symptoms and laboratory sampling was longer in this group (*P* < .001), and they were less often advised antiviral treatment (*P* < .001), however, declined treatment less. The highest cumulative incidence was seen in the West Midlands region (32.7/10 000), London (7.0/10 000) and East of England region (5.7/10 000).

**Conclusions:**

People of South Asian ethnic groups were disproportionally affected by the first wave of the influenza pandemic in England in 2009. The findings presented contribute to further understanding of demographic, socioeconomic and ethnic factors of the outbreak and inform future influenza preparedness to ensure appropriate prevention and care.

## 1 INTRODUCTION

The first cases of pandemic influenza A(H1N1)pdm09 were reported in Mexico in March 2009. The following month imported cases were reported in the United Kingdom. Rapid global spread resulted in the first influenza pandemic for 40 years and the second recorded H1N1 pandemic.^1^ The first wave of the 2009 pandemic in the UK peaked in late July and the second wave peaked in late October, with an estimated 780 000 case of infection,^2^ of whom 474 had died up to April 2010.^3^

Greater pandemic‐related morbidity and mortality were reported for indigenous and minority ethnic groups in several countries including the UK.^4^, ^5^, ^6^, ^7^, ^8^, ^9^, ^10^ Sociodemographic variation in the impact of infectious diseases can arise due to differences in exposure, susceptibility and/or treatment; there may be language and cultural barriers to seeking and receiving appropriate care.^11^

The South Asian ethnic group (UK residents of Indian, Pakistani or Bangladeshi birth or heritage) is one of the largest ethnic minority groups in England, accounting for 5.6% of the total population in the 2011 national census.^12^ During the UK pandemic, there was an overrepresentation of South Asian ethnic groups among hospital admissions and deaths (including paediatric deaths) associated with pandemic influenza.^9^, ^13^, ^14^ This study aims to ascertain if this overrepresentation also existed in the overall number of laboratory‐confirmed pandemic influenza cases in England suggesting greater exposure or susceptibility in this population rather than greater severity of illness (eg due to co‐morbidities).

The study aimed to estimate the occurrence of influenza A(H1N1)pdm09 infections in South Asian populations, compared to other ethnic group populations, during the first phase of the 2009 influenza pandemic in England. At this time, all new cases coming to the attention of public health and primary care services underwent laboratory testing, to improve understanding of possible social differences in the impact of influenza infection and inform preparedness for future pandemics.

## 2 METHODS

The study is based on laboratory data obtained during the 2009 pandemic. In the “containment” phase of the public health response (from April to July 2009), suspected influenza H1N1pdm09 cases (both ambulatory and hospitalised) were routinely tested for infection, confirmed cases were treated with antivirals, and contacts were traced and offered post‐exposure antiviral chemoprophylaxis. A “treatment” phase (from July 2009) started once sustained community transmission occurred, where all suspected cases were treated with antivirals without laboratory confirmation and contact tracing was discontinued.^15^

Data on laboratory‐confirmed cases of influenza A(H1N1)pdm09 in England were collected by the Health Protection Agency using “FluZone”, a centralised online case management system used for capturing demographic and clinical information on cases, between April and October 2009. Ethnic group data in FluZone were largely incomplete but, as full names of all cases were available, the computerised South Asian Names and Group Recognition Algorithm (SANGRA) was used to impute the missing data. The SANGRA algorithm can identify if a name is likely to be of South Asian origin, that is from the Indian subcontinent. The development and validation of the method are described elsewhere^15^ ^;^ it has been used in several studies lacking complete ethnic group data.^16^, ^17^, ^18^ The limited data on ethnic group recorded in FluZone were compared to the imputed data to estimate the sensitivity and specificity of the SANGRA algorithm.

Personal data were handled in line with strict Public Health England information security guidelines. This analysis was undertaken under the Health Service (Control of Patient Information) Regulations SI1438/2002 which provide a basis for collecting and processing data without patient consent for the purposes of communicable disease control in England. As this was part of public health surveillance, no ethical approval for this analysis was required or sought.

Mid‐year population estimates from 2009 by region and ethnic group were obtained from the Office of National Statistics (ONS).^19^ Available postcode data were geocoded, matched with corresponding 2010 area‐level Indices of Multiple Deprivation (IMD) scores^20^ and subsequently divided into IMD quintiles, with 1 being least deprived to 5 being most deprived.

Epidemic curves by week of onset were plotted showing case counts stratified by ethnic group (South Asian or other). For cases with a missing onset date, the date of onset was imputed by subtracting two days from the sample date (based on the median observed difference of 2 days between onset date and sample date for the other cases).

Cases in each group are summarised in terms of age, sex, deprivation, region of residence, underlying health conditions, pregnancy and antiviral provision. To test for statistical differences, chi‐square tests and t tests were performed as appropriate. No information on outcome of infection was available. Rates per 10 000 population for South Asian and other cases were calculated by region; we calculated rate ratios to compare rates. A log‐rank test was undertaken to determine significance of date of clinical sample versus symptom onset by ethnic group.

A point map was created showing the geographical distribution of cases of South Asian and other ethnicities in England. Clustering of South Asian ethnic cases was compared with clustering of other cases in London and the West Midlands (the regions with the highest number of cases) using Ripley's K functions. Ripley's K function is a technique used to assess if individual geographical points are dispersed, clustered or randomly distributed within a given area by assessing whether the average number of cases within a given distance of another case is statistically greater than expected from a random spatial distribution, and was used here to assess differences in geospatial clustering between South Asian and other cases. K functions are calculated at multiple distances to examine whether clustering differs depending on the scale used.^21^

**Figure 1:**
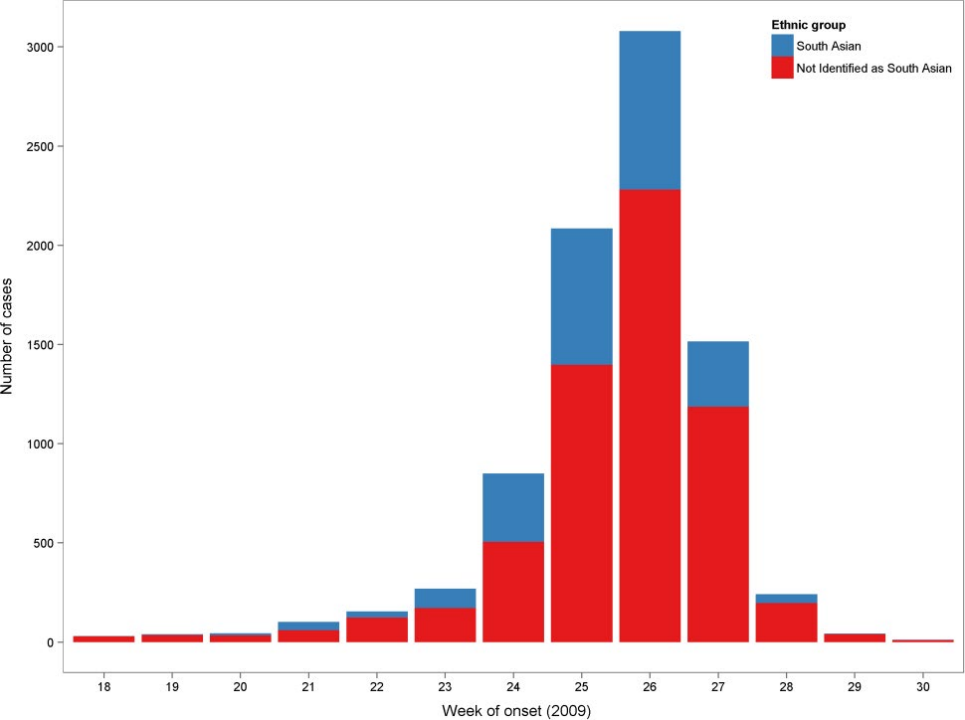
Number of confirmed cases of influenza A(H1N1) pdm09 by week of onset* and ethnic group, England, 2009 (n=8466**)

Analyses were performed using R (version 3.1.1).^22^

## 3 RESULTS

Between April and October 2009, 8748 confirmed cases of influenza A(H1N1) were recorded on the FluZone surveillance system. Data fields were not completed for all confirmed cases—where there were key missing data (such as postcode or date of onset), these records have been excluded from analysis.

Ethnic group was recorded for 636 cases (7.3%). Data imputed by the SANGRA algorithm were compared to available ethnic group data, showing that 169 of 211 South Asian cases were correctly identified by the algorithm (sensitivity of 80%, 95% CI: 76.6%‐83.6%) and 323 of 337 of known non‐South Asian ethnic group (specificity of 96%, 95% CI: 94.6%‐97.2%) were correctly classified. Based on the combined ethnic group data, 2447 (28%) confirmed cases were considered to be of South Asian ethnic group for this analysis.

The first cases were reported in week 18 of 2009 (week beginning 27 April) and the number of recorded cases increased exponentially in the following weeks (Figure 1). The highest number (3080 cases) was recorded in week 26, after which the number of records declined to less than 50 in week 29 as a result of progression to the treatment phase and a fall‐off in data collection. The last case was recorded in week 42.

**Figure 2:**
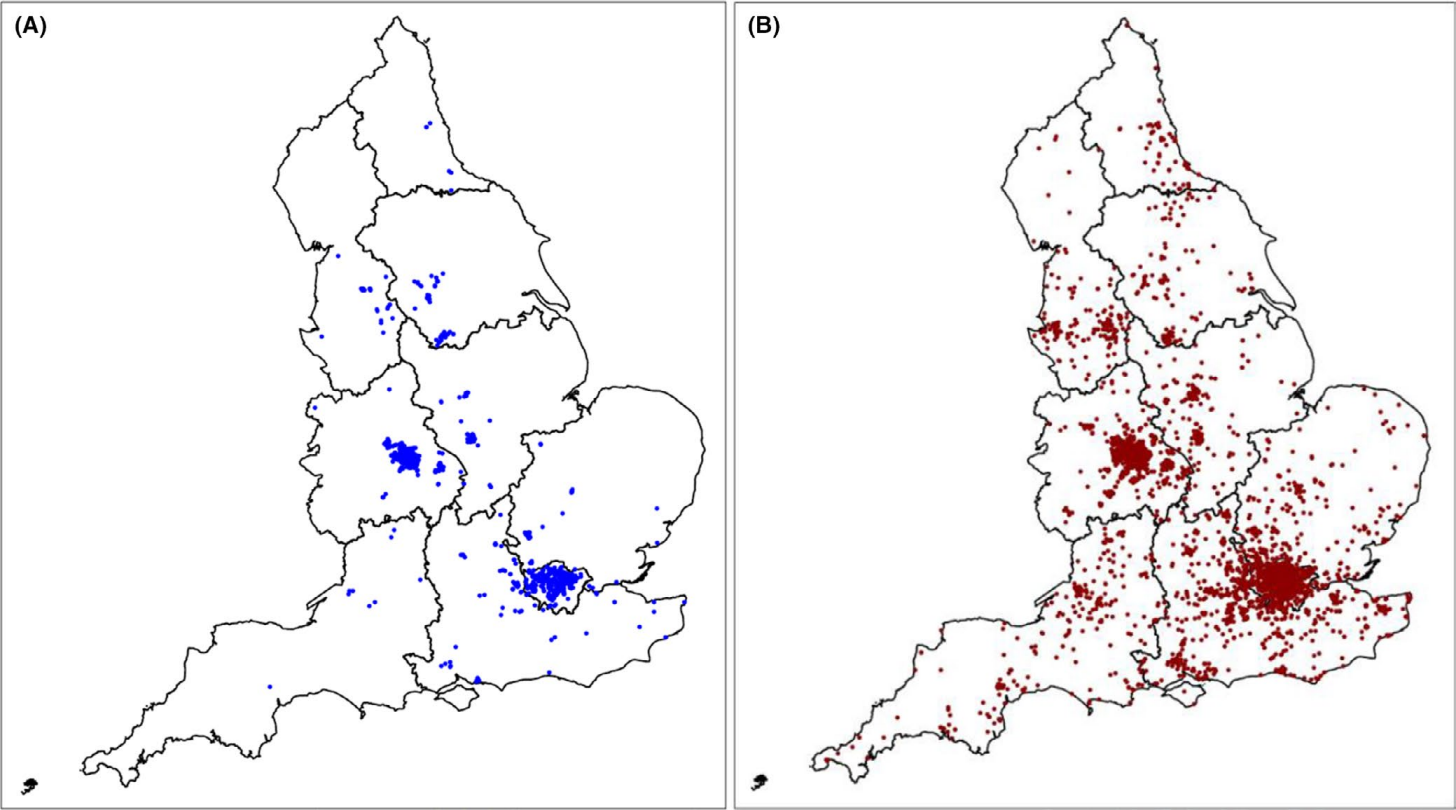
A, Geographical distribution of South Asian cases of influenza A(H1N1) pdm09, England, 2009. B, Geographical distribution of other cases of influenza A(H1N1) pdm09, England, 2009

*

**

A slight male predominance was seen overall (51%) which was more pronounced among cases of South Asian ethnic groups (54%) than among cases of other ethnic groups combined (50%, *P* = .002, Table 1). Overall, age ranged from 0 to 90 years with a median age of 14 years. Cases of South Asian ethnic group were younger compared to all other groups; their median age was 12 years, and more than half of the cases were in the 5 to 14 years age group. For other ethnic groups, the median age of cases was significantly higher at 16 years (*t* test; *P* < .001) and the highest percentage of cases was in the 15 to 49 years age group.

**Table 1:** Characteristics of cases by ethnic group, England, 2009

	All cases n (%)	Cases of South Asian ethnic group n (%)	Cases of other ethnic group n (%)	*P*‐value (test performed)
Gender (n = 8287)				
Male	4256 (51.4)	1254 (54.1)	3002 (50.3)	.002 (*χ* ^2^ test)
Female	4031 (48.6)	1063 (45.9)	2968 (49.7)	
Age (n = 8587)				
Under 5 y	642 (7.5)	261 (10.7)	381 (6.2)	<.001 (*t* test)
5‐14 y	3700 (43.1)	1255 (51.4)	2445 (39.8)	
15‐49 y	3878 (45.2)	853 (34.9)	3025 (49.2)	
50‐64 y	310 (3.6)	60 (2.5)	250 (4.1)	
65 y and above	57 (0.7)	13 (0.5)	44 (0.7)	
National IMD (n = 7086)				
5 (most deprived)	3941 (55.6)	1649 (78.5)	2292 (46.0)	<.001 (*χ* ^2^ test)
4	1035 (14.6)	264 (12.6)	771 (15.5)	
3	863 (12.2)	111 (5.3)	752 (15.1)	
2	507 (7.2)	33 (1.6)	474 (9.5)	
1 (least deprived)	740 (10.4)	43 (2.0)	697 (14.0)	
Underlying conditions (n = 8748)				
No reported co‐morbidities	8699 (99.4)	2462 (99.6)	6165 (99.4)	.346 (χ2 test)
Chronic respiratory disease	26 (0.3)	5 (0.2)	21 (0.3)	
Pregnancy	28 (0.3)	12 (0.5)	16 (0.3)	
Diabetes	7 (0.1)	0 (0)	7 (0.1)	
Immunosuppression	9 (0.1)	3 (0.1)	6 (0.1)	
Chronic heart disease				
Chronic liver disease	Results suppressed due to low patient numbers (<0.1% for all indicators)			
Chronic renal disease				
Antiviral provision (n = 5637)				
Advised	4201 (74.5)	1140 (69.7)	3061 (76.5)	<.001 (*χ* ^2^ test)
Declined	130 (2.3)	22 (1.3)	108 (2.7)	
Not advised	1306 (23.2)	474 (29.0)	832 (20.8)	

More than three‐quarters of the cases of South Asian ethnic group were from the most deprived national IMD quintile areas (78%), significantly more than for cases of other ethnicities (46%; *P* < .001). ONS data are not readily available to undertake combined age and IMD stratification of cases.

Limited clinical information was available. An underlying medical condition was reported for only 49 cases, mainly chronic respiratory disease (n = 26), immunosuppression (n = 9) and diabetes (n = 7). There were no significant differences in underlying medical conditions between the two groups. Pregnancy was reported in 12 South Asian cases and 12 cases of other ethnicities.

There were significant differences in antiviral treatment provision to cases of South Asian ethnic group in comparison with cases of other ethnic group (*P* < .001); twenty‐nine per cent of South Asian cases were recorded as not having been advised to have antiviral treatment compared to 20.8% of all other ethnic groups. The percentage of cases declining treatment was slightly lower among South Asians than those from other ethnicities (1.3% vs 2.7%).

A valid postcode of residence was available for 7094 of all cases (81%). The distribution of South Asian and other cases across England is shown in Figure 2A,B. The maps are consistent with large clusters of intense influenza transmission in urban areas, mainly in the two largest cities of England, London and Birmingham in the West Midlands (Figure 2) in the early phase of the pandemic in the UK. Few cases had been reported from outside these areas, especially South Asian cases.

**Figure 3:**
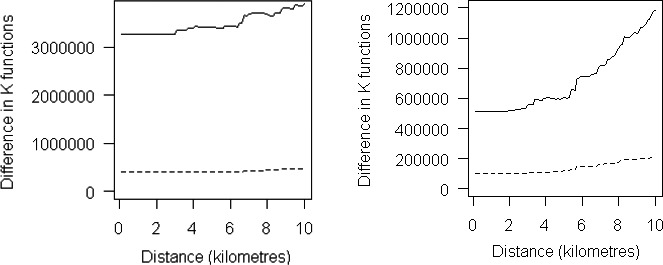
A, K functions comparing the spatial distribution of South Asian and other cases of influenza A(H1N1) pdm09, London 2009. B, K functions comparing the spatial distribution of South Asian and other cases of influenza A(H1N1) pdm09, West Midlands 2009

Figure 3 shows the difference in K functions between South Asian and other cases in London and the West Midlands over a range of distances. The dotted line represents the null hypothesis of no difference in clustering/dispersion.^23^ The line exceeding the envelope represented by the dashed lines in the K function charts indicates that spatial clustering is significantly greater in cases of South Asian ethnic group compared to other cases.

The South Asian proportion of the total population is highest in London region (11.1%) followed by the West Midlands region (7.8%) (Table 2). The highest cumulative incidence of confirmed influenza A(H1N1) pdm09 infection for South Asian cases was seen in the West Midlands region (32.7/10 000) followed by London (7.0/10 000) and East of England (5.7/10 000). For England, the relative risk of laboratory‐diagnosed infection among South Asian cases compared to cases of other ethnic group was 6.8, with the highest relative risks in the West Midlands (10.4), Yorkshire & the Humber (8.8), East of England (6.1) and the East Midlands (5.5).

**Table 2:** Population numbers and cumulative incidence of laboratory‐confirmed influenza H1N1pdm09 infection region and ethnic group, England, 2009

Region	Total population	South Asian population (% of total)	Number of reported South Asian cases (crude rate/10 000 population)	Number of reported other cases (crude rate/10 000 population)	Relative risk (South Asian: Other)	95% CI for Relative Risk
East Midlands	4 451 200	216 300 (4.9)	55 (2.5)	195 (0.5)	5.5	5.2‐5.8
East of England	5 766 600	219 600 (3.8)	125 (5.7)	515 (0.9)	6.1	5.9‐6.3
London	7 753 600	863 100 (11.1)	608 (7.0)	2370 (3.4)	2.0	2.0‐1.9
North East	2 584 300	59 900 (2.3)	6 (1.0)	66 (0.3)	3.8	2.9‐4.6
North West	6 897 900	293 700 (4.3)	30 (1.0)	167 (0.3)	4.0	3.6‐4.4
South East	8 435 700	311 100 (3.7)	117 (3.8)	824 (1.0)	3.7	3.5‐3.7
South West	5 231 200	103 300 (2.0)	8 (0.8)	369 (0.7)	1.1	0.4‐1.8
West Midlands	5 431 100	423 500 (7.8)	1385 (32.7)	1575 (3.1)	10.4	10.3‐10.5
Yorkshire & Humber	5 258 100	298 300 (5.7)	83 (2.8)	156 (0.3)	8.8	8.5‐9.0
Unknown Region	‐	‐	74	19	‐	‐
England	51 809 700	2 789 100 (5.4)	2532 (8.7)	6216 (1.3)	6.8	6.7‐6.8

The time from onset of symptoms to laboratory sampling was significantly longer for South Asian cases than for other cases (log‐rank test chi‐squared value 30.6; *P*=<0.001).

## 4 DISCUSSION

Over a quarter (28%) of laboratory‐confirmed cases of influenza A(H1N1)pdm09 recorded in the central pandemic influenza case management system in England between April and October 2009 were identified as belonging to a South Asian ethnic group based on recorded and SANGRA‐imputed ethnic group data. The relative risk of influenza A(H1N1)pdm09 for the South Asian population compared to the remaining population was 6.8, indicating that disproportionally high numbers of laboratory‐confirmed pandemic influenza cases of South Asian ethnic group were reported.

Comparing the imputed ethnic group data identified by this method to the ethnic group data reported by a subset of the cases, we estimated a sensitivity of 80% and a specificity of 96% for the SANGRA method. Even allowing for misclassification of 4% of other ethnic groups, the overrepresentation of cases in the South Asian population will still be apparent.

The overrepresentation of South Asian cases is in line with the results of previous studies carried out in the UK. In a study of 631 patients with confirmed infection admitted to hospitals, 31.5% (169/631) were of Asian or Asian British ethnic group during the first wave^14^ and 21.8% over both waves (249/1140).^24^ Mandatory reporting of suspected and confirmed deaths of pandemic influenza showed that mortality rates were higher for Indian cases (aIRR 1.87) and Pakistani cases (aIRR 3.37) in England than those of the White British ethnic group.^9^ A study describing laboratory‐confirmed cases in the West Midlands identified 57.9% as South Asian cases by manual classification,^25^ a striking proportion that is higher than the amount estimated by this study.

In the early pandemic, following multiple importations local transmission was mainly found in the urban areas of London and the West Midlands. These areas accounted for two‐thirds of the overall number of cases and over three‐quarters of South Asian cases reported. Urban residents are initially possibly more exposed to the pandemic influenza than rural residents as there is higher mobility, higher population density and a more diverse population. Much of the transmission was driven by school‐aged children with outbreaks reported in school settings.^26^ Urban areas in the United States were also more affected by pandemic influenza than rural areas.^27^ In a previous UK study, crude analysis showed that the risk of mortality was higher for urban cases; however, this association was absent after adjustment for deprivation.^9^

Relatively, high proportions of the London and West Midlands populations comprise of South Asians (11.1% and 7.8% respectively) compared to the national average (5.4%). According to our findings, South Asian cases were also significantly more spatially clustered than other cases in urban centres. Compared to the average household size of 2.4, Bangladeshi, Pakistani and Indian households are considerably larger with respective average household sizes of 4.5, 4.1 and 3.3^28^ which, together with a younger and more susceptible population,^27^, ^28^ may further contribute to transmission of influenza.

The percentage of cases classified as being in the most deprived quintile areas was high at 56%, providing further evidence that transmission is associated with deprivation as previously found in a study describing early cases in London.^29^ Reflecting observations in the general population, cases of South Asian ethnic group resided significantly more often in the most deprived quintile areas (79%) than other cases (46%); however, this percentage was lower in South Asian cases attending hospitals in England (26%)^20^ possibly as a result of different referral practices or differences in severity of disease. Deprivation has also been associated with higher mortality among influenza cases previously.^9^, ^30^

Just under half of all cases were younger than 15 years (46%). The high percentage of children being affected by pandemic influenza differs from typical seasonal influenza, where the vast majority of cases are found in the older age groups; this result was also observed in other countries such as the United States^27^ and Canada.^31^ Compared to cases of other ethnic groups, more South Asian cases belonged to younger age groups. This can partly be explained by the younger age profile of the South Asian population in England: 22% were younger than 15 years in 2009, vs 17% in other ethnic groups.^19^ This difference was not found among influenza A(H1N1)pdm09 cases admitted to hospital.^14^ However, it was apparent in a study on paediatric mortality due to influenza in England that mortality rates for Bangladeshi and Pakistani children under 18 years of age (36 and 47 deaths per million from June 2009 to March 2010, respectively) were substantially higher compared to White British children (4 deaths per million).^13^

In addition, the difference in antiviral treatment advice between South Asian and other cases is unexplained and its implications for impact on South Asian groups are unclear. Antiviral treatment was advised significantly less often for South Asian cases (*P *= <0.001). There were no apparent differences in pregnancy or underlying health conditions between the groups; however, limited incomplete data were available. Differences in antiviral treatment could arise from delayed clinical presentation or laboratory sampling in South Asian groups (our analysis provides some evidence for the latter); language barriers; and/or poorer access to healthcare services in areas with high South Asian populations.

The strength of this study is that individual case information was available from a large number of laboratory‐confirmed cases across England, including data on geographical location. The well‐validated SANGRA method^15^, ^32^ was used to impute ethnic group based on case names where data on ethnic group were incomplete. The sensitivity found in this study is lower than in previous studies using the SANGRA method where sensitivity was 89%‐96%,^15^, ^32^ this could have led to underestimation of found differences such as for geographical clustering. Reported ethnic group was only available for 7.3% of cases and was therefore a small number to validate the method from. The specificity was high which is in line with previous reported specificity of 94%‐99%.^15^, ^32^

The main limitation from this method is that only South Asian ethnic groups can be identified. South Asian names are distinctive and can therefore more easily be identified by a computerised algorithm.^15^ The cases not identified as South Asian therefore comprises all other ethnicities residing in England which might combine groups with different characteristics. However, our data suggest South Asian ethnic group is associated with deprivation and urban residence, all of which have been previously associated with increased impact from the pandemic. Differences in health‐seeking behaviour between the two groups could have been another explanatory factor for the high proportion of South Asian cases, but this could not be assessed by this study.

Data were limited to the early phase of the first wave of the pandemic, and therefore does not give a complete view of the number of cases during the two pandemic waves, particularly given that not all cases were laboratory confirmed during either wave.^33^

This study adds further evidence that South Asians had excess incidence of influenza A(H1N1)pdm09 in 2009 in England and that demographic and geographical characteristics of South Asian cases differ from other cases. A younger age profile as well as being mostly from urban, more deprived areas and less often being offered antiviral treatment are risk factors previously identified with higher risk of acquiring pandemic influenza and were more found among South Asian cases which has probably contributed to this overrepresentation. The results contribute to the understanding of the demographic, socioeconomic and ethnic factors relating to the pandemic influenza in 2009 in England.

We recommend that future preparedness and response for outbreaks of influenza and other respiratory viruses such as COVID‐19 takes account of these outcomes by addressing the needs of ethnic minorities through removing barriers to preventive care and treatment, and ensuring appropriate culture‐specific communications. Routine recording of data on ethnic group and other known risk factors would result in more complete data and would allow for tailored recommendations for the different ethnic groups residing in the country.

## CONFLICT OF INTERESTS

The authors declare that they have no competing interests.

## AUTHOR CONTRIBUTION

**Suzan C. M. Trienekens:** Data curation (equal); Formal analysis (equal); Methodology (equal); Visualization (equal); Writing‐original draft (lead); Writing‐review & editing (equal). **Wendi Shepherd:** Data curation (supporting); Formal analysis (supporting); Visualization (supporting); Writing‐original draft (supporting); Writing‐review & editing (equal). **Richard G. Pebody:** Conceptualization (supporting); Data curation (supporting); Methodology (supporting); Software (equal); Validation (equal); Writing‐original draft (supporting); Writing‐review & editing (supporting). **Punam Mangtani:** Conceptualization (supporting); Data curation (supporting); Methodology (supporting); Software (equal); Validation (equal); Writing‐original draft (supporting); Writing‐review & editing (supporting). **Paul Cleary:** Conceptualization (lead); Data curation (equal); Formal analysis (equal); Methodology (equal); Supervision (lead); Visualization (equal); Writing‐original draft (supporting); Writing‐review & editing (equal).

## Footnotes

* 30 cases were reported after week 30.

** For 252 cases, no onset date or sample date were available.
